# Fast on–off controlling of electrohydrodynamic printing based on AC oscillation induced voltage

**DOI:** 10.1038/s41598-023-30956-7

**Published:** 2023-03-07

**Authors:** Huatan Chen, Junyu Chen, Jiaxin Jiang, Zungui Shao, Guoyi Kang, Xiang Wang, Wenwang Li, Yifang Liu, Gaofeng Zheng

**Affiliations:** 1 Pen-Tung Sah Institute of Micro-Nano Science and Technology, Xiamen University, Xiamen, 361102 China; 2 Shenzhen Research Institute of Xiamen University, Shenzhen, 518000 China; 3 School of Mechanical and Automotive Engineering, Xiamen University of Technology, Xiamen, 361024 China

**Keywords:** Materials for devices, Nanoscale materials, Structural materials

## Abstract

Stability control of electrohydrodynamic (EHD) printing technology is urgent needed for efficient fabrication of flexible electronics. In this study, a new fast on–off controlling technology for micro droplets of EHD is proposed by applying an AC induced voltage. The suspending droplet interface is broken through quickly, and the impulse current can be significantly reduced from 527.2 to 50.14 nA, which greatly reduces its negative impact on jet stability. What’s more, time interval of jet generation can be shortened by a factor of three, while not only significantly improving the uniformity of the droplets, but effectively reducing the droplet size from 195 to 104 μm. Moreover, the controllable and mass formation of micro droplets are realized, but also the structure of each droplet is able to be controlled independently, which promoted the development of EHD printing technology in more fields.

## Introduction

With the continuous development of flexible electronic and soft robots, some innovative materials have higher requirements in toughness^1^, thermal conductivity^2,3^ and other aspects. In recent years, the application of liquid materials in flexible electronics in the form of micro droplets to improve various properties has attracted extensive attention because of its simple preparation process, good compatibility, superior performance, and mutual isolation^4–10^. For example, liquid metal (LM) is a good material because of its good electrical, thermal and mechanical properties, which is suitable for electrical, thermal and other functionalities flexible devices^11,12^. Many researchers proved that the liquid metal materials such as LM have great application prospects, however, it is still necessary to further develop manufacturing methods, and produce micro droplets of different sizes and shapes to improve the preparation efficiency and enhance the tenability and control of functional properties^13^.

Electrohydrodynamic (EHD) printing technology is one of the main methods for fabrication of micro droplets, which deform the viscous solution into a Taylor-cone for the printing of micro/nano structures by an external electric field^14,15^. The printing frequency, the average diameter, and the uniformity of droplets has always been important factors in EHD printing^16–18^. Rehmani and Arif^19^ presented a methodology which highlighted the importance of nozzle shape to govern smaller droplets even with a large head diameter, Vu et al.^20^ introduced the usage of a 55 degrees chamfered nozzle in an EHD system to control stability of single-jet mode and enhanced the production quality. Both DC voltage and AC voltage source are suitable for jet printing. Nguyen and Byun^21^ used AC pulse voltage for EHD printing and proved that it can overcome the defect of electrical breakdown caused by DC voltage. However, in the process of micro droplets made by EHD technology, it is difficult to achieve a rapid transformation between "jetting mode" and "stopping state" in adjacent cycles when the jet is about to be formed. The process can’t be stopped or started immediately according to the demand in a short time under the condition of ensuring the morphology and accurate positioning of the micro droplets. The reason is the surface of the meniscus reacts quickly under the electric field but also hard to be controlled, which means that the initial droplet must be abandoned when the starting voltage is applied, and only continuous droplet generation process can be realized, in other words, instantaneous on–off cannot be achieved.

Typically, the electric fields are created by establishing a pulse voltage difference between the nozzle and the collector in EHD, deforming the Taylor cone and eventually leading to instability that results in droplets produced from the apex of the cone^14,22–25^. In the initial stage of applying the pulse voltage, the instability caused by large impulse voltage will lead to intense oscillation of the suspended droplets at the nozzle, and the jet will take away a larger volume of solution, which means the uniformity of droplets may be negatively affected^26–28^. In the previous works of our group, it has been proved that the use of AC power could more effectively suppress the negative effects of charge accumulation, and the sharp edge of the voltage wave is likely to cause instability in the early stages of ejection process^29,30^.

In this paper, a new EHD technology was proposed by applying an AC induced voltage (named AC oscillation induced voltage) that was not enough to break through the interface of suspended droplet before the pulse voltage, so that made it store more energy than that without one, which meant less energy need to be absorbed by suspended droplet for producing the charged jet. The rapid breakthrough of the suspended droplet interface and formation of charging jets were realized, and the instability of the jet could be effectively reduced. Some teams have put forward similar ideas about assisted starting of printing. Rahman et al.^31^ tried to add DC step voltage to assist the starting process of electro-hydraulic jet printing, and use three-stage pressurization method to improve the stability of jet printing. Li et al.^32^ proposed an optimized waveform design to quickly eliminate the residual oscillation in EHD DOD printing by adding an additional voltage pulse when the jet stops and retracts. The method we proposed is based on the AC voltage. The charge polarity of the suspended droplet is in the process of dynamic conversion. Under the condition that the droplet can accumulate energy, and also can maintain a more dynamic and stable state of the volume. The influence of the electric field force on the suspended droplet can be much smaller. Not only the uniformity of the micro droplets is significantly improved, but the structure and morphology of each droplet were controlled independently. The new method enables micro droplets to have broader prospects for applications in more fields.

## Materials and methods

### System device

**Figure 1:**
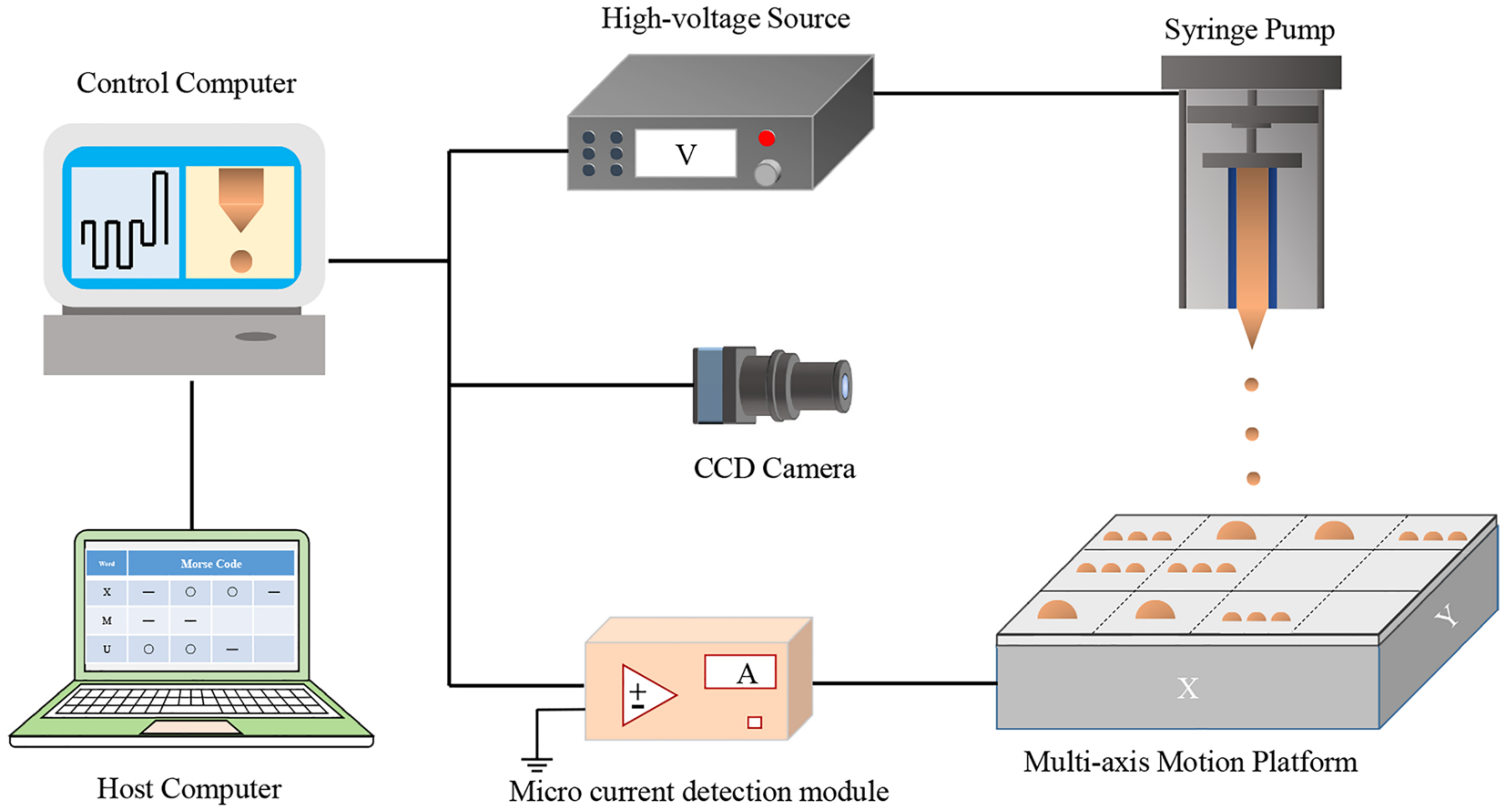
Electrohydrodynamic printing experimental system.

An experimental system for EHD was built as shown in the Fig. 1, including a host computer, a control computer, a high-voltage source (RIGOL DG1022Z, Beijing, China; HVA-502NP5, Tianjin, China), a CCD camera (uEye UI-3130CP-C-HQ, IDS Imaging Development Systems GmbH, Obersulm, Germany), a precision syringe pump (Pump 11 Pico Plus Elite, Harvard Apparatus America, Cambridge, MA, USA), and a multi-axis motion platform (REI95LM-050, Shenzhen Borui Automation Equipment Co., Ltd., Shenzhen, China). The controllable high-voltage power was connected to the spinneret, creating a high-voltage electric field between the spinneret and the collector. The solution was supplied to the spinneret by the precision syringe pump. A silicon wafer was used as the collector and fixed on the multi-axis motion platform.

### Materials

The concentration of 4 ωt% Polyethylene oxide (*Mw* = 300,000 g/mol, Guangdong TPL Chemical Co. Ltd, China, the solvent was a mixture of deionized water and ethanol with volume ratio of 3:1 (*v*_*w*_*:v*_*e*_)) mixed with a concentration of 10 ωt% Silver nanoparticles (S18870, Shanghai Acmec Biochemical Co., Ltd China) was prepared, and a Rhodamine dye with a mass fraction of 2% were added to improve the observability of the jet and droplets.

### Characterization and measurements

The morphology of micro droplets was recorded by the microscope (OLYMPUS CX31, Japan) and the size was analyzed by ImageJ. The change of micro-current in the experimental process was observed by a self-designed current detection module, which can be combined with the suspended droplet at the nozzle photographed by CCD camera for further analysis.

## Results and discussions

### Interface analysis and critical conditions for charged jet generation

First of all, the interface fluctuations and critical conditions generated by the charged jet need to be clarified. The ejection of the charged jet is a coupled process of multiple physical fields^30^. Under the action of high-voltage electric field, the fluid is subject to the joint action of electric field force, viscous force, gravity and surface tension to produce rheological behaviors such as oscillation, tension, refinement and fracture. The starting stage of charged jet mainly depends on the force characteristics and charge accumulation behavior of droplets at the nozzle^33,34^.

**Figure 2:**
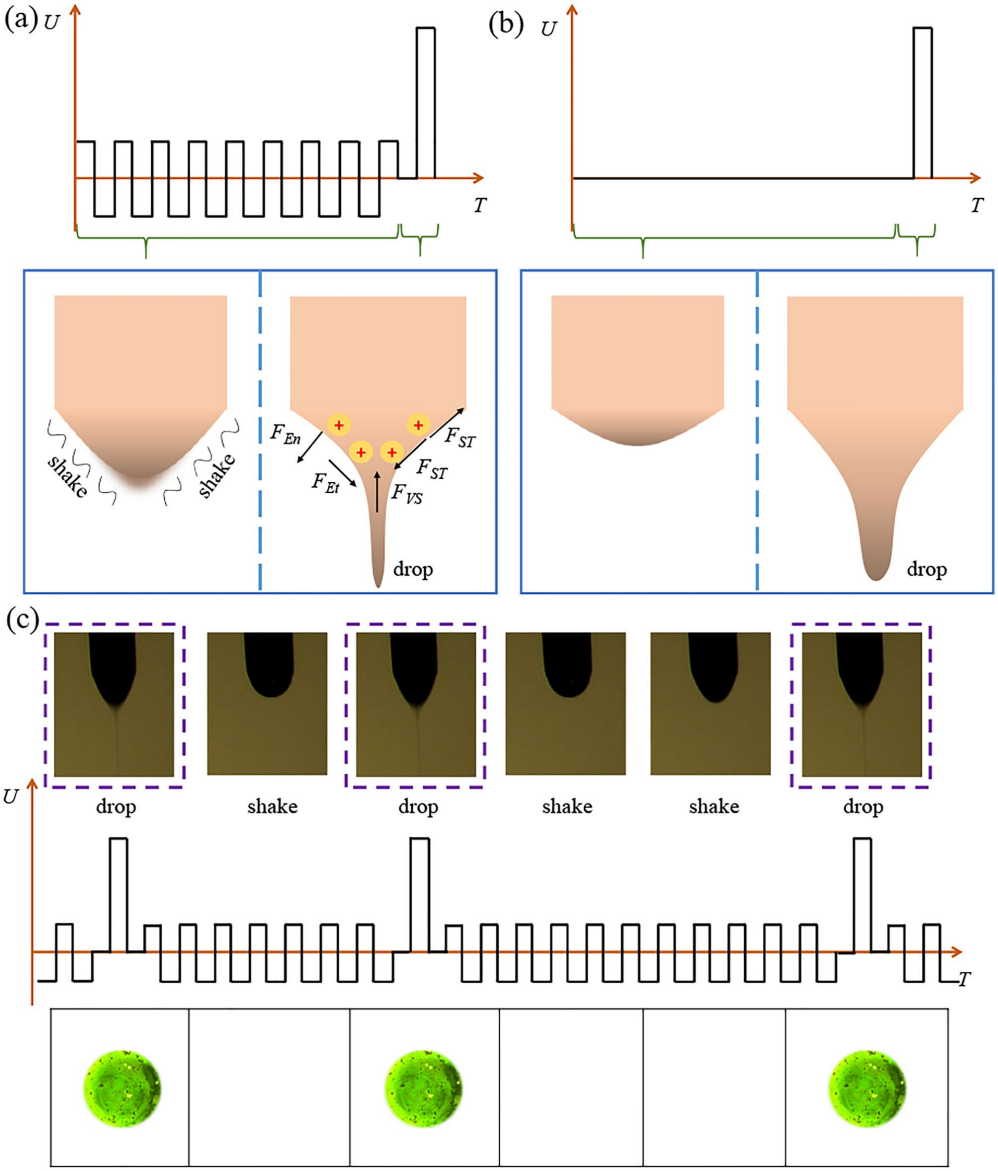
(**a**) Effect of AC induced voltage and pulse voltage on suspended droplet. (**b**) Effect of pulse voltage on suspended droplet. (**c**) Sequence diagram of matching among voltage, jet and droplet.

“Leaky dielectric model” is used usually to describe the process of charged fluid^35,36^. As shown in the Fig. 2a, the rheological dynamic model of EHD is constructed. The Navier–Stokes equation can describe the fluidity of incompressible viscous fluid in space high voltage electric field^37^.

1 $$ \rho \left[ {\frac{{\partial \mathop{v}\limits^{\rightharpoonup} _{i} }}{\partial t} + \mathop{v}\limits^{\rightharpoonup} _{i} \cdot\nabla \mathop{v}\limits^{\rightharpoonup} _{i} } \right] = - \nabla p_{i} + \mathop{F}\limits^{\rightharpoonup} _{ESi} + \mathop{F}\limits^{\rightharpoonup} _{VSi} + \mathop{F}\limits^{\rightharpoonup} _{STi} + \rho_{i} \mathop{g}\limits^{\rightharpoonup} $$

In this equation, $\rho$ is the fluid density, $v$ is the fluid velocity, *p* is the hydrostatic pressure (i.e. thermodynamic pressure), $\nabla p$ is the differential pressure force between two phases, $\mathop{F}\limits^{\rightharpoonup} _{ES}$ is the electric field force, $\mathop{F}\limits^{\rightharpoonup} _{VS}$ is the viscous force, $\mathop{F}\limits^{\rightharpoonup} _{ST}$ is the surface tension of two-phase interface, and $i$ is the fluid phase.

And the electric field force $\mathop{F}\limits^{\rightharpoonup} _{{ES{ }}}$ can be deduced by the following formula:2

The $\overleftrightarrow T^{M}$ is the Maxwell stress tensor, *q* is the density of space charge at the interface, and $\mathop{E}\limits^{\rightharpoonup}$ is the space electric field Strength, ε is the dielectric constant of the fluid. Therefore, we can conclude that $\mathop{F}\limits^{\rightharpoonup} _{ES}$ and q show a certain proportional relationship^38,39^.

Therefore, the influence of the electric field force on the suspended droplets can be controlled by affecting the charge, which proved the effective of the AC induced voltage. The AC induced voltage that is not enough to break through the interface of suspended droplet before the pulse voltage was used, as shown in the Fig. 2a. The sequence diagram of matching among voltage, jet and droplet is shown in the Fig. 2c, in the stage of AC induced voltage, the suspended droplet will vibrate continuously because of the change of charge polarity and electric field force. In the stage of pulse voltage, the electric field force will overcome the surface tension, and the surface of polymer solution will be break through and droplets can be achieved. In contrast, when the jet is started by a pulse voltage, as shown in Fig. 2b, the generation of the jet often carries more volume of solution, because it needs to absorb more energy to reach the critical condition, and larger impulse voltage may produce greater fluctuations when breaking through the surface interface of the suspended droplet.

**Figure 3:**
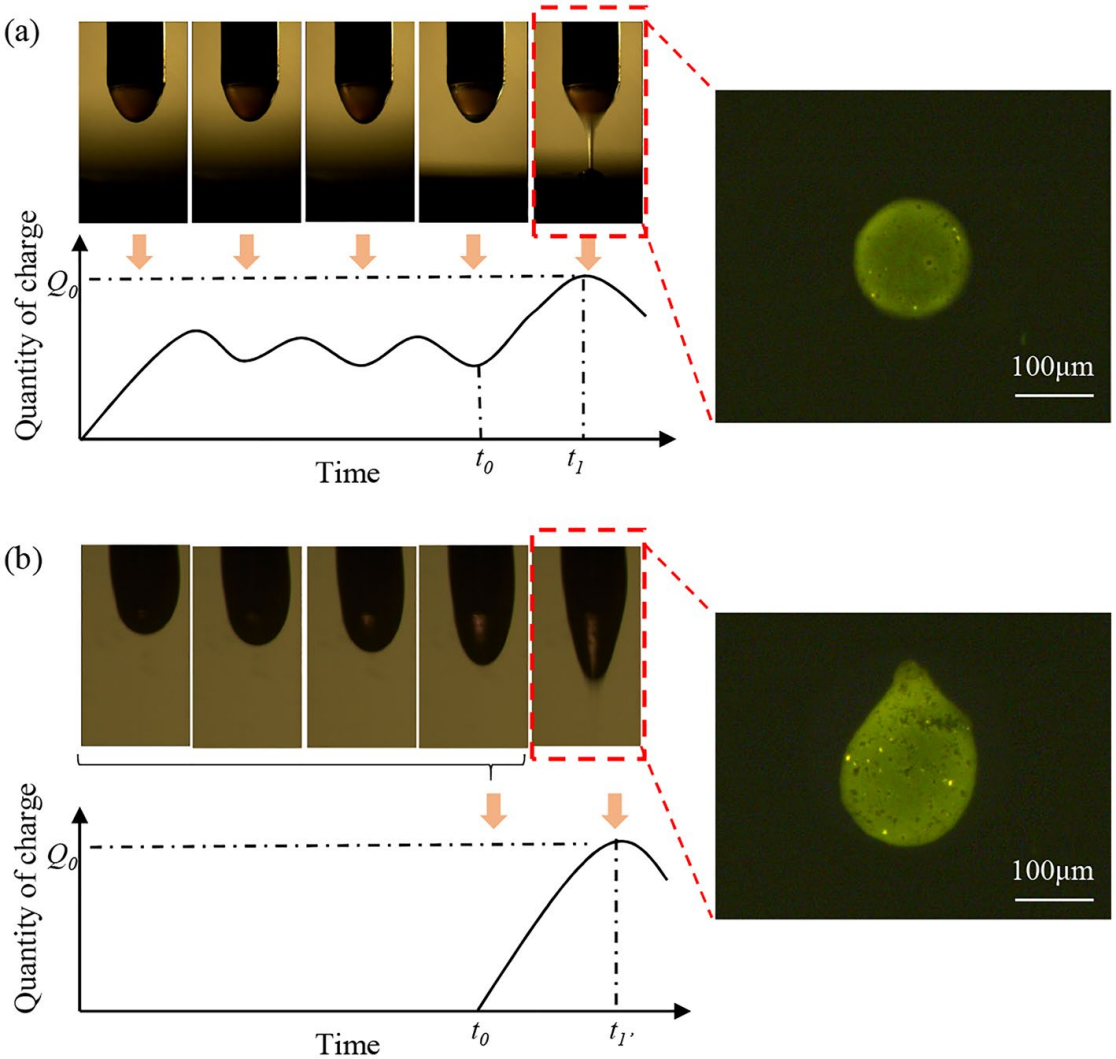
The starting stage of jet generated. (**a**) Applied AC oscillation induced voltage for the jet generation. (**b**) Applied pulse voltage for the jet generation.

Generally speaking, EHD often uses pulse voltage to generate electric field and form charged jet^4,32,40^. The process of charge accumulation is shown in the Fig. 3, *t*_0_ is the moment when the pulse voltage is applied, *Q*_0_ is the critical charge required for the suspended droplet to produce the jet ejection, and *t*_1_/*t*_1′_ are the moment corresponding to the jet ejection. The charge of the suspended droplet accumulates rapidly when the voltage generates an electric field. When the critical charge is exceeded, it means that the electric field force overcomes the surface tension, and then the jet is generated.

When the AC voltage which is unable to produce charged jet used before *t*_0_ (named AC oscillation induced voltage), the charge on the suspended droplet is in a process of continuous accumulation and neutralization, as shown in the Fig. 3a. The reason for this phenomenon is that the AC field is different from the pulse voltage, which the alternating polarity between positive and negative will make the charge of suspended droplet in a trend of increasing and then decreasing. According to formula we have mentioned, we can find the $\mathop{F}\limits^{\rightharpoonup} _{ES}$ will also be effected by the change of charges, which will occur that the force of the suspended droplet vary over time, just like a spring of continuous compression and relaxation.

However, when pure pulsed voltage is applied, the charge on the suspended droplet presents a dramatic increase trend until the critical charge *Q*_0_ is exceeded. By comparing Fig. 3a, b, it can be concluded that *t*_1_ is usually less than* t*_1′_, because in terms of charge accumulation, the suspended droplet that applied AC voltage has accumulated amount of charge, which also means that it can be reached the critical amount of charge in less time. Moreover, the jet can be started with less energy to reduce the instability caused by the sharp edge of the impact voltage, which indicates that the whip generated when the jet is formed into droplets can be effectively inhibited, thus ensuring the morphology of the collected droplets. At the same time, it can also be clearly seen from the contrast diagram of the jet that the Taylor cone under the AC induced voltage has a larger cone angle, and the volume of micro droplets have been taken away from the suspended droplet in smaller volumes, which makes it possible to further reduce the size of micro droplets.

### Effect of applied voltage on jet starting process

**Figure 4:**
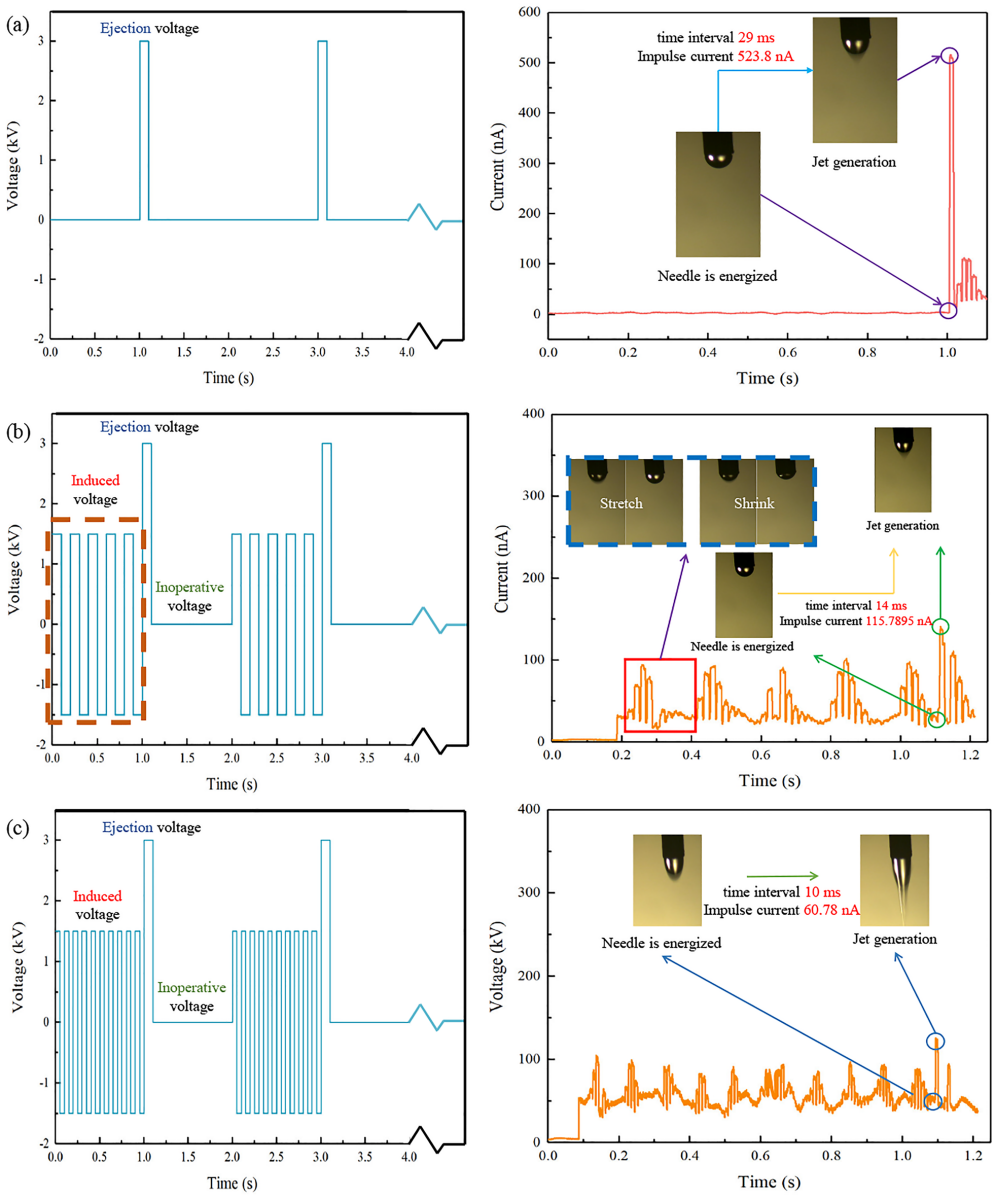
The waveform of the applied voltage and micro-current detection. (**a**) Pulse voltage. (**b**) 5 Hz AC induced voltage and pulse voltage. (**c**) 10 Hz AC induced voltage and pulse voltage.

The current detection module cooperates with the CCD camera for the EHD, and the applied voltage used as the variable. The pulse voltage control group is shown in the Fig. 4a, which the pulse voltage is applied at the moment of 1 s, which corresponds to the peak current captured in the current sequence diagram. At the moment of applying the voltage, the suspended droplets stretch abruptly to form Taylor cone, the time interval of produce the charged jet is 29 ms, and the impulse current generated is as high as 527.1 nA.

The AC oscillation induced voltage is used under the same experimental conditions, as shown in the Fig. 4b, the applied voltage includes three parts: induced voltage, ejection voltage and inoperative voltage. The suspended droplet showed a stretching-shrink cycle regularly captured by CCD camera under the condition of 5 Hz AC induced voltage, and the frequency of stretching-shrink cycle is consistent with the voltage frequency. The time between the needle has been energized and the generation of charged jet is 14 ms, which is twice shorter than that under the condition of pulse voltage without induced. Simultaneously, compared with the impulse current of pulse voltage without induced, it is also reduced by four times. And Fig. 4c proved that this decreasing trend will be further reflected with the increase of induced voltage frequency.

The trend of time interval of jet generation and impulse voltage under induced voltage of different frequencies is recorded in Table 1 and Fig. 4. The influence of AC induced voltage on Taylor cone oscillation before jetting has been researched. The change of the space electric field will cause the change of the dielectric constant at the fluid interface, which will produce the dielectric force perpendicular to the two-phase interface, and make the suspended drop produce forced vibration. It can be concluded from the Fig. 4 and Table that the increase of the AC oscillation induced voltage frequency will increase the Taylor cone oscillation frequency. When the AC induced voltage frequency is small, the Taylor cone oscillation frequency is close to the voltage frequency. However, with the acceleration of charge polarity conversion, the response frequency of Taylor cone will be affected by the charge relaxation time, which is different from the voltage frequency. The increase of the oscillation frequency of the Taylor cone can effectively reduce the impulse current from 527.1 to 50.14 nA or even lower. The reason for this phenomenon is the induced voltage makes the suspended droplet keep in reserve more energy, so the energy required to achieve jet ejection will be greatly reduced, which means that it plays a significant role in improving the stability of the jet start stage. At the same time, the time required for jet generation can be also greatly reduced, which make the function of rapid on–off can be realized.

**Table 1:** Effect of induced voltage frequency on jet initiation.

Frequency of the induced voltage (Hz)	Time interval of jet generation (ms)	Taylor cone oscillation frequency	Impulse current (nA)
0	29.3	/	527.1
5	14.5	5	115.8
25	10.4	24	60.78
50	10.2	47	55.54
75	9.3	70	53.74

### Effect of induced voltage on droplet morphology

**Figure 5:**
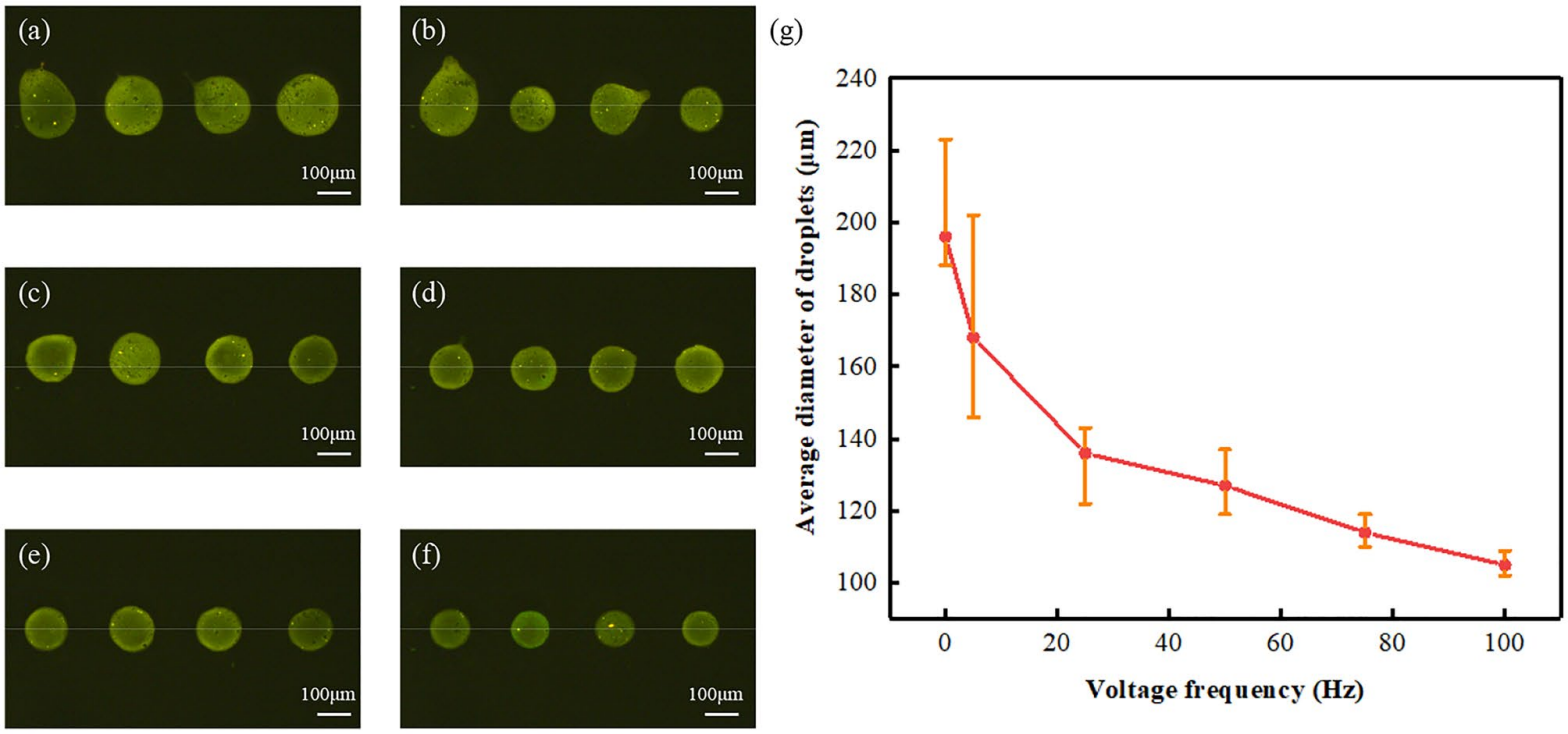
(**a**)–(**f**) The average diameter of the droplets with different frequency of induced voltage (**a**) 0 Hz, (**b**) 5 Hz, (**c**) 25 Hz, (**d**) 50 Hz, (**e**) 75 Hz, (**f**) 100 Hz. (**g**) The relationship between size of the droplets and the frequency of applied induced voltage.

The average size of the droplets in EHD was observed by microscope under the condition of different frequency induced voltage, as shown in Fig. 5a–f. The relationship between the average diameter of droplets and the voltage frequency is recorded in Fig. 5g, which can be concluded that the average size of the droplets will decrease significantly from 195 to 104 μm with the increase of induced voltage frequency. The reason for this relationship is the use of AC oscillation induced voltage, the additional energy required to break through the solution interface is reduced, in the meantime, the effective improvement of impulse current can also avoid the instability caused by sharp edges of the voltage. As a result, in the process of the droplets break through the limit of suspended droplets at the nozzle, the volume of solution taken away is greatly reduced, thus the average size of droplets is inversely proportional to the frequency of the induced voltage. In addition, we can also find from the distribution of the error bar that the effective improvement of the impulse current can greatly reduce the instability of the droplets when the jet is generated, which has a key improvement on the quality of the generation of EHD droplets.

In the experiment, the printing frequency of the droplet depends on the frequency of the pulse voltage. Therefore, the used of induced voltage will prolong the time interval of micro droplets and reduce the printing frequency. The relationship between the frequency of the pulse voltage and the print droplet is also explored. When the frequency of the pulse voltage is less than 2 Hz, the printing frequency is consistent with the frequency of the pulse voltage. However, when the frequency of the pulse voltage is greater than 2 Hz, there will be multiple liquid droplets in a single cycle, or satellite liquid droplets, or even not reaching the critical point of injection.

**Figure 6:**
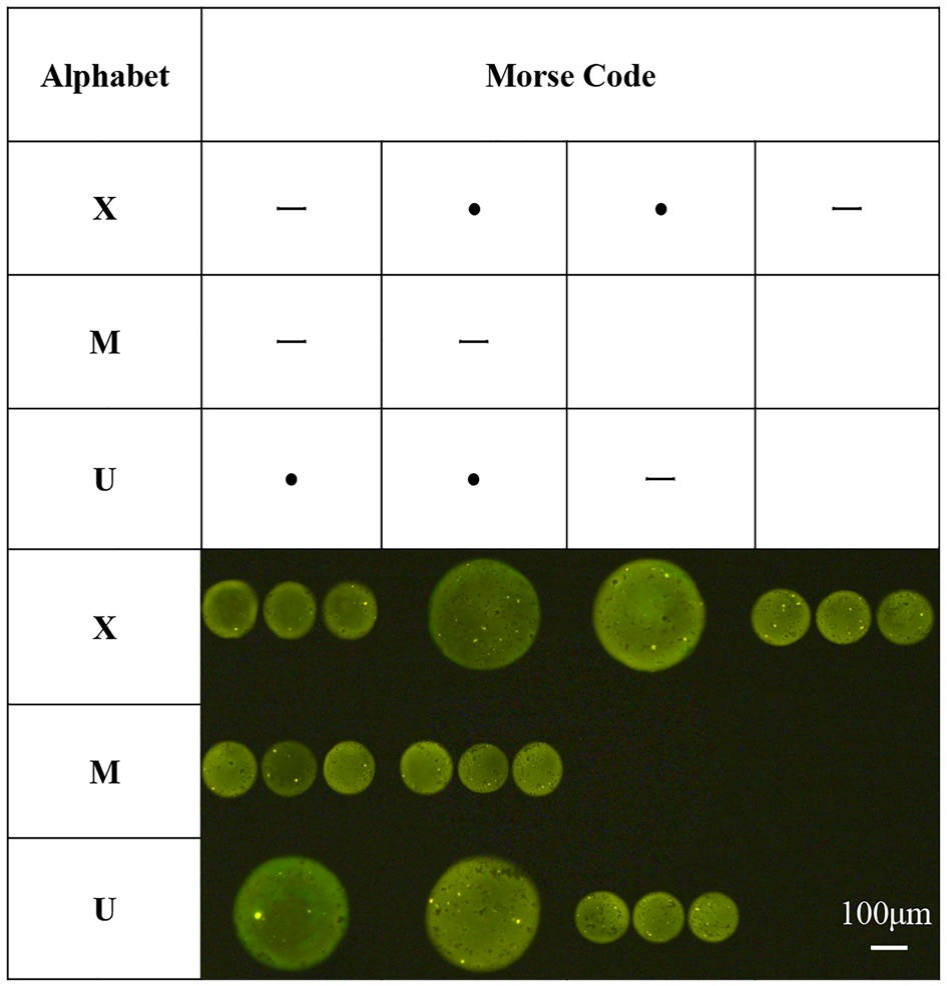
Morse code corresponding to alphabet and micro droplets represent Morse code by different size.

To verify whether each droplet can be controlled independently, this technology has been used to achieve the preparation of Morse code, as shown in Fig. 6. The dots and lines in Morse code are simulated by distinguishing the number and size of droplets. Draw a 4*3 grids on the silicon substrate, and each four grid represents the Morse code of a letter. Three small droplets with a diameter of about 120 μm were located in one grid representing the “line” in the Morse code by means of the fast on–off controlling EHD printing which we recommend. Then the size of the droplets was controlled by modifying the process parameters, and the droplets with a diameter of about 290 μm were located accurately in the grid representing the “point” in the Morse code. Therefore, the independent control of each droplet structure is completed by using the fast on–off controlling EHD printing technology, and achieved the simulation experiment of Morse code.

## Conclusions

In this study, a new technology of fast on–off controlling of EHD printing is proposed by applying an AC oscillation induced voltage that is not enough to break through the interface of suspended droplet before the pulse voltage, to make it store more energy than that without AC oscillation induced voltage, which means less energy need to be absorbed by suspended droplet for producing the charged jet. The reduction of impulse current and the time interval of jet generation can not only solve the problem that not every droplet can be applicable, but improves the uniformity and order of droplets achieved on the collector, which provides the possibility for the structure of each droplet can be controlled independently.

## Data Availability

The authors confirm that the data supporting the findings of this study are available within the article.
